# Supplementary material for: Evidence of Isomerization in the Michael-Type Thiol-Maleimide Addition: Click Reaction between L-Cysteine and 6-Maleimidehexanoic Acid
Source: Molecules. 2022 Aug 9;27(16):5064. doi: 10.3390/molecules27165064 (PMC9415311; doi:10.3390/molecules27165064)
Supplement: Supplementary file 1 [file molecules-27-05064-s001.zip › molecules-1808289-supplementary.pdf]

Supplementary Materials

Evidence of Isomerization in the Michael-Type Thiol-Maleimide Addition: Click Reaction Between L-Cysteine and 6-Maleimidehexanoic acid.

Víctor Alfonso Niño Ramírez [1], Diego Sebastián Insuasty Cepeda [1], Zuly Jenny Rivera Monroy[1*] and Mauricio Maldonado

Table of Contents:

1. **Figures:**

Figure S1. ^{1}H-NMR spectrum (400MHz, MeOD, 303 K) of compound **1**.

Figure S2. ^{13}C-NMR spectrum (400MHz, MeOD, 303 K) of compound **1**.

Figure S3. MS/MS for TIC of 4.7 min corresponding to compound **1**.

Figure S4. ^{1}H-NMR spectrum (400MHz, MeOD, 303 K) of compound **2**.

Figure S5. ^{13}C-NMR spectrum (400MHz, MeOD, 303 K) of compound **2**.

Figure S6. ^{1}H-NMR COSY spectrum (400MHz, MeOD, 303 K) of compound **2**.

Figure S7. ^{1}H-NMR HSQC spectrum (400MHz, MeOD, 303 K) of compound **2**.

Figure S8. ^{1}H-NMR HMBC spectrum (400MHz, MeOD, 303 K) of compound **2.**

Figure S9. MS/MS for TIC of 4.6 min corresponding to compound **2**.

Figure S10. ^{1}H-NMR spectrum (400MHz, MeOD, 303 K) of mix products.

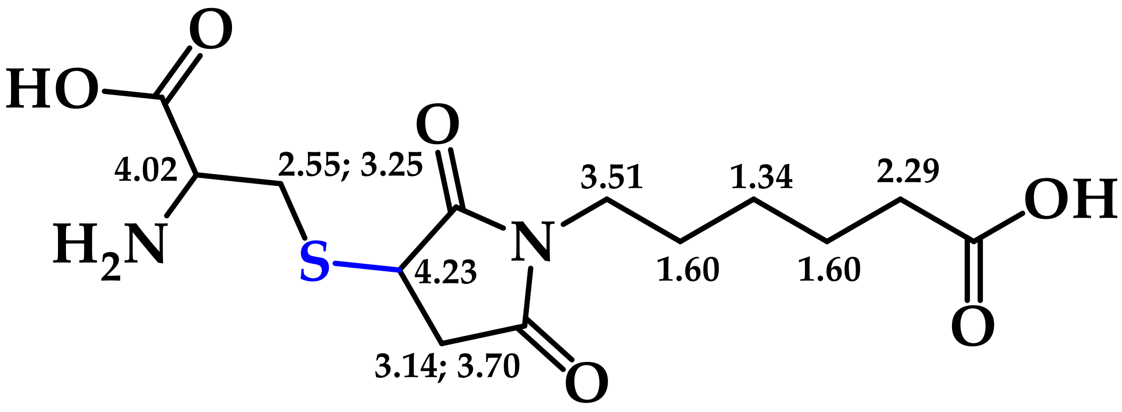

COMPOUND 1: Cys-S-*Mhx*

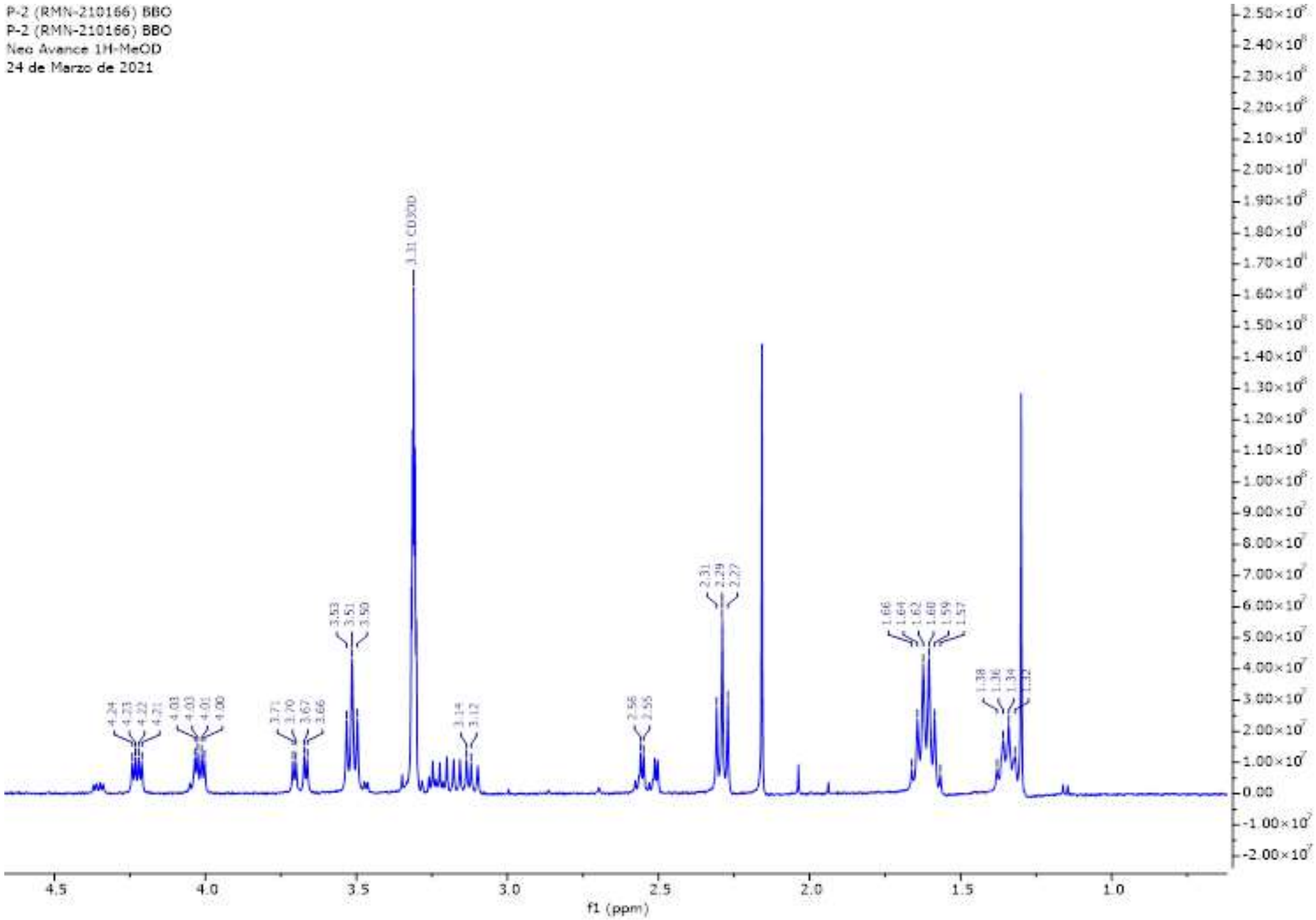

Figure S1. ^{1}H-NMR spectrum (400MHz, MeOD, 303 K) of compound **1**.

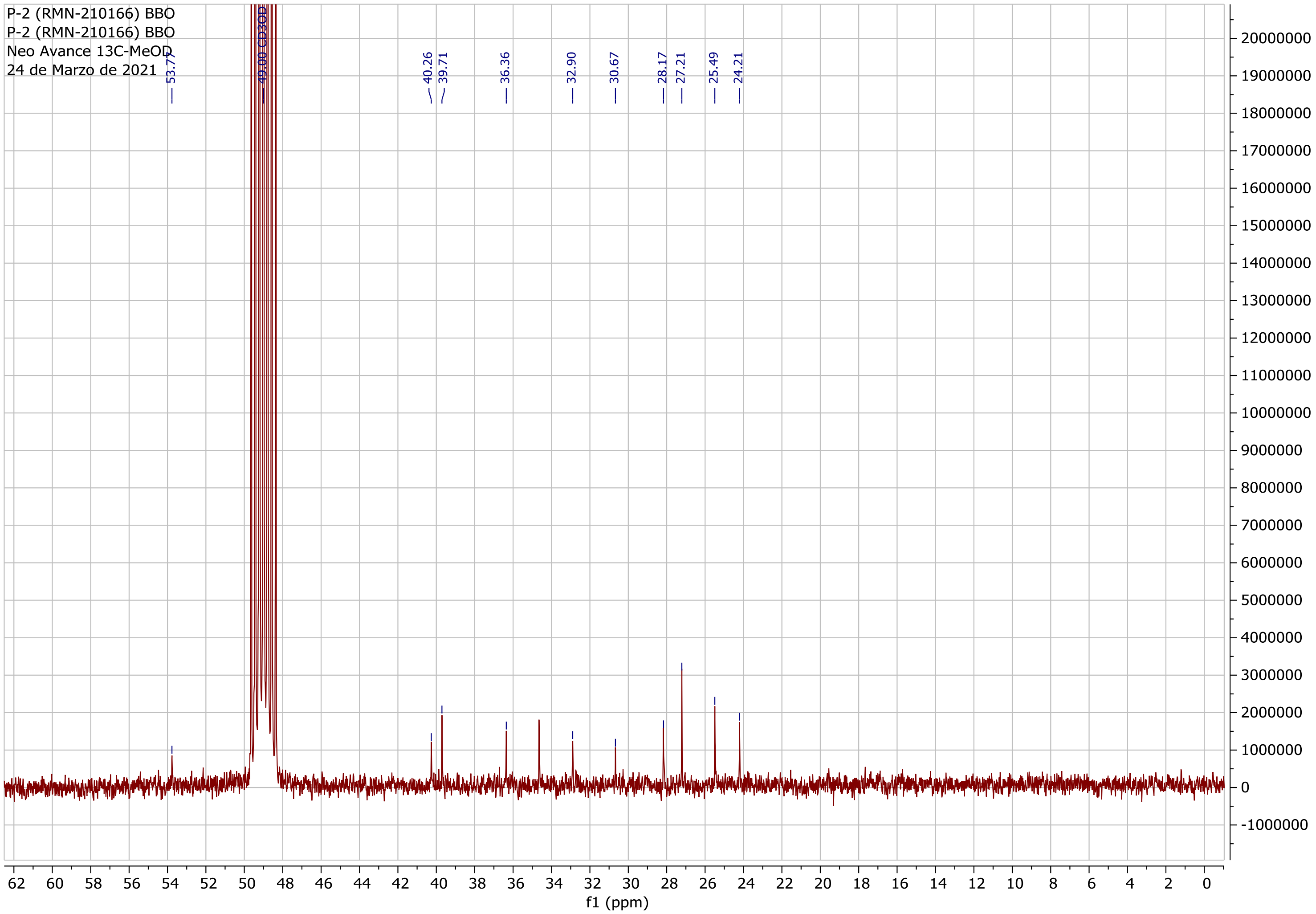

Figure S2. ^{13}C-NMR spectrum (400MHz, MeOD, 303 K) of compound **1**.

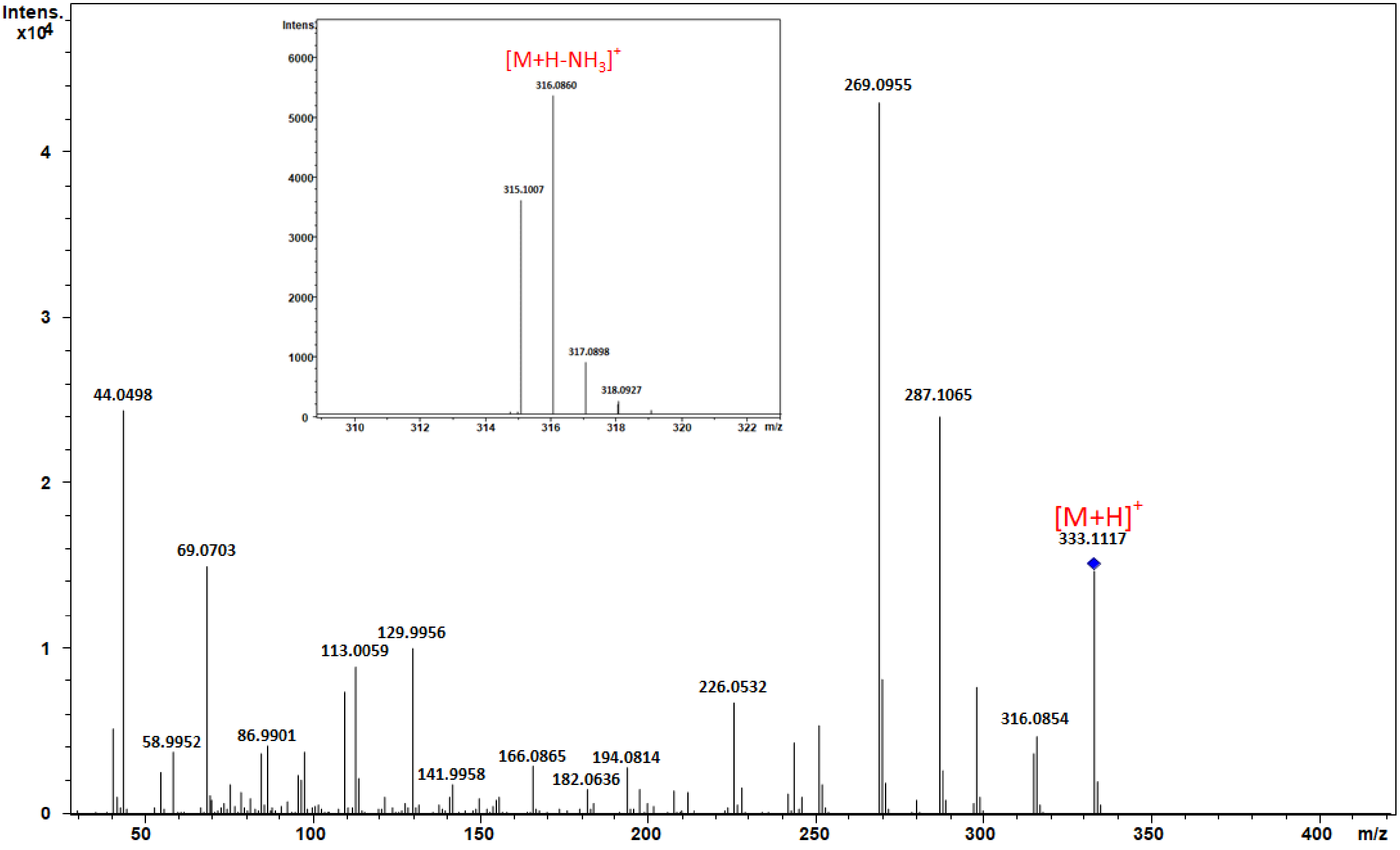

Figure S3. MS/MS for TIC of 4.7 min corresponding to compound **1**.

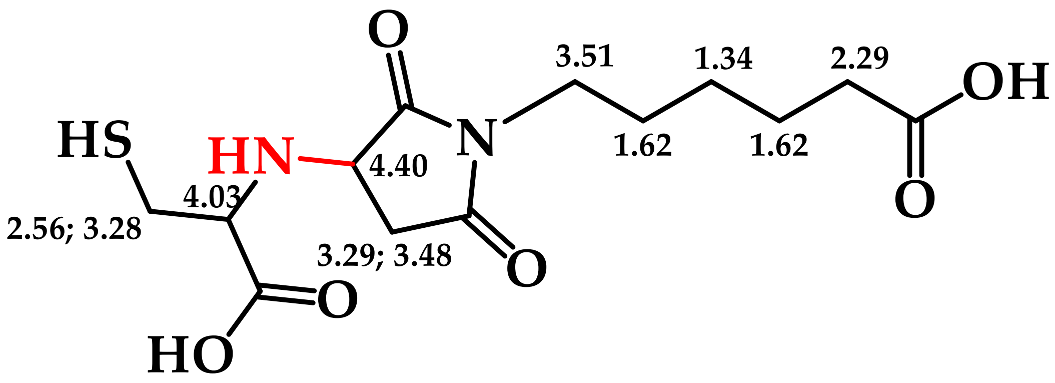

COMPOUND 2: Cys-N-*Mhx*

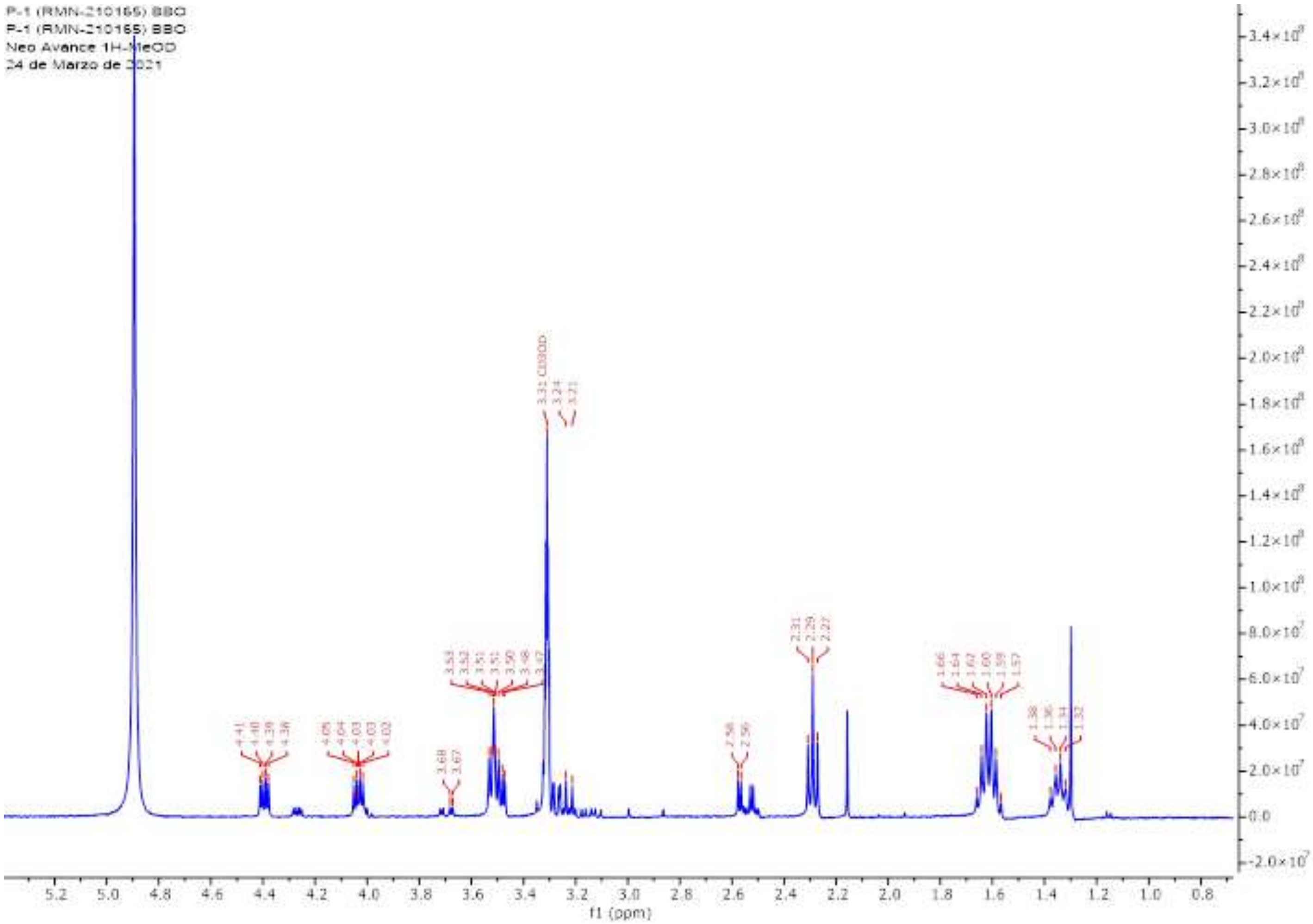

Figure S4. ^{1}H-NMR spectrum (400MHz, MeOD, 303 K) of compound **2**.

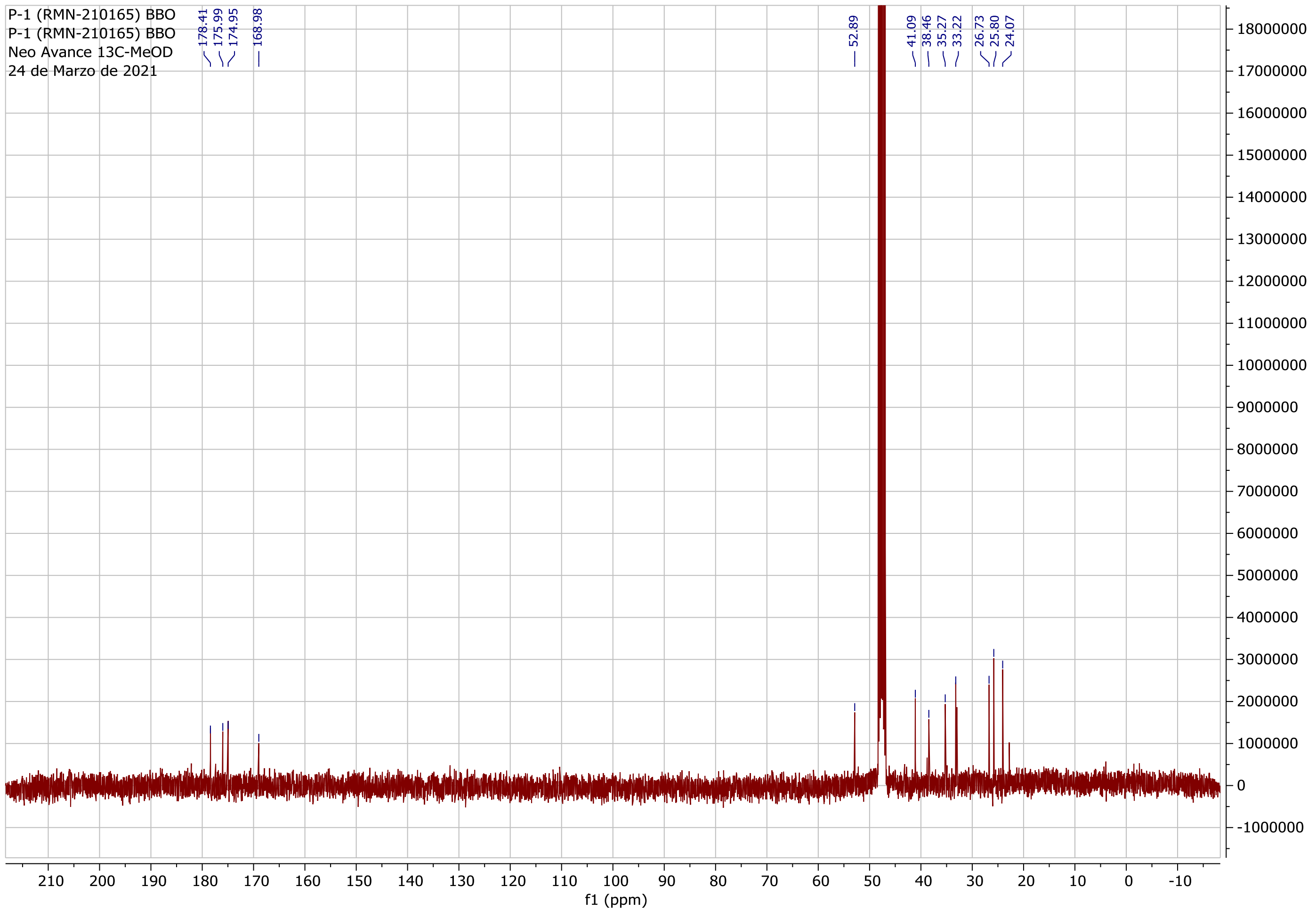

Figure S5. ^{13}C-NMR spectrum (400MHz, MeOD, 303 K) of compound **2**.

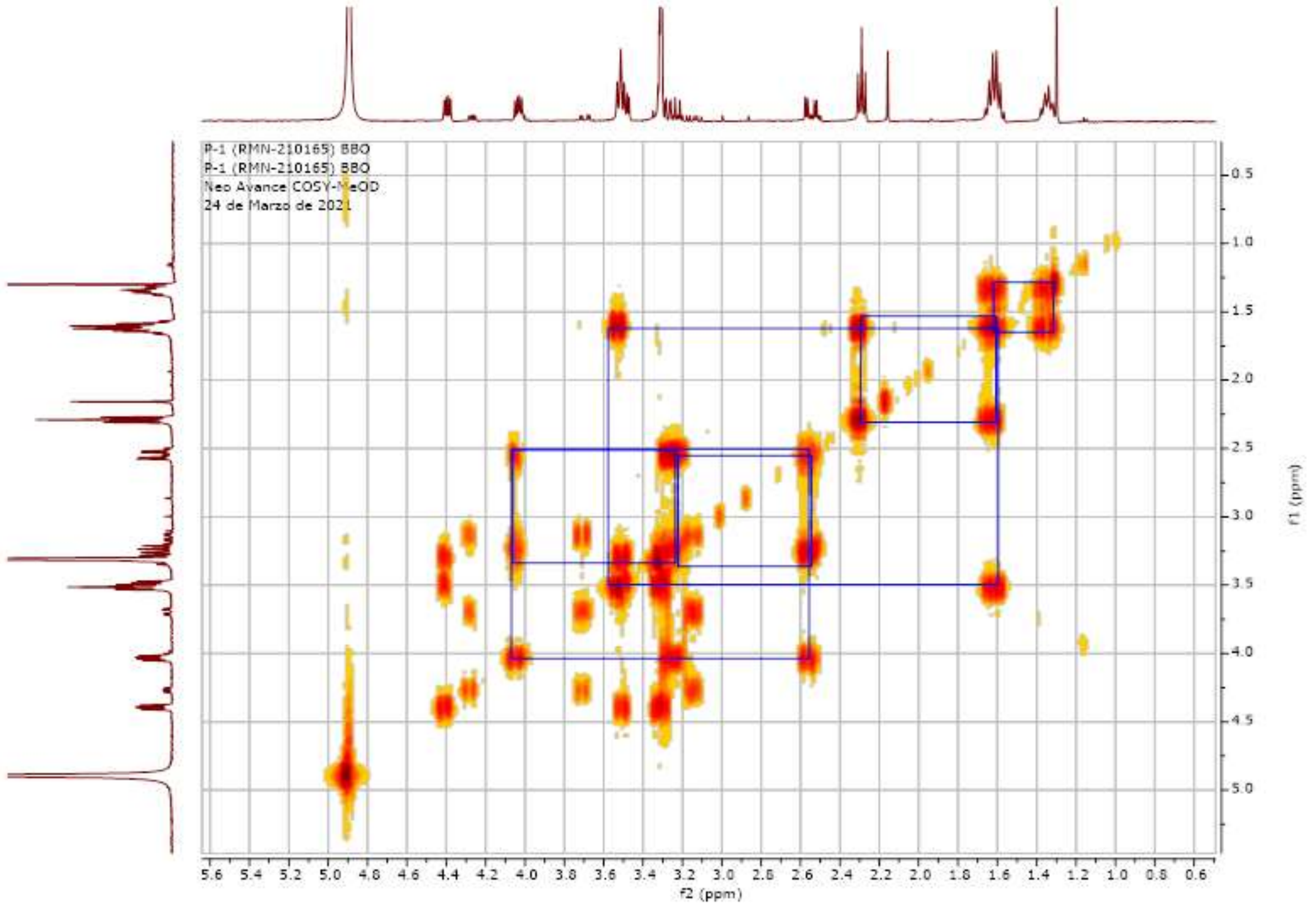

Figure S6. ^{1}H-NMR COSY spectrum (400MHz, MeOD, 303 K) of compound **2**.

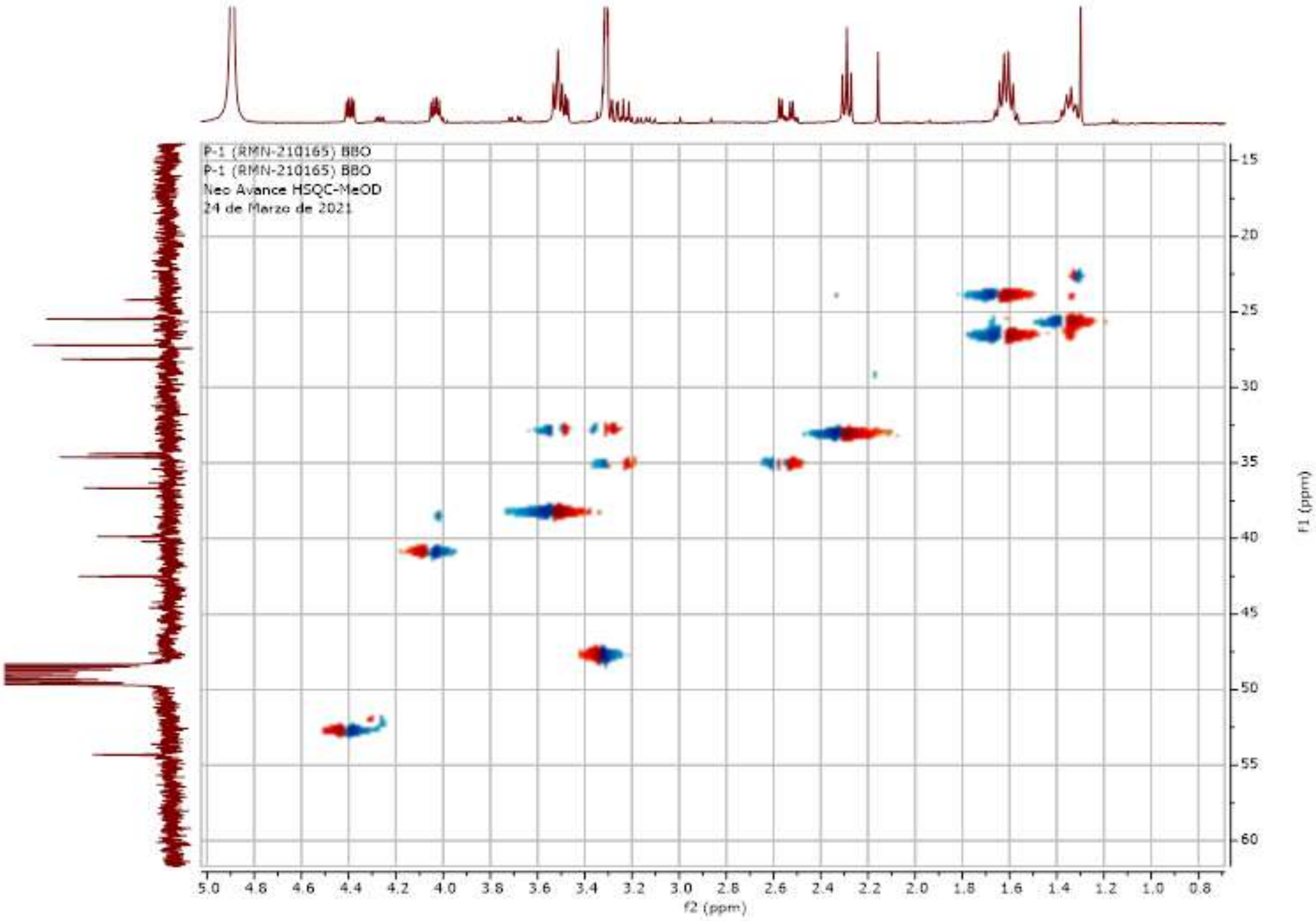

Figure S7. ^{1}H-NMR HSQC spectrum (400MHz, MeOD, 303 K) of compound **2**.

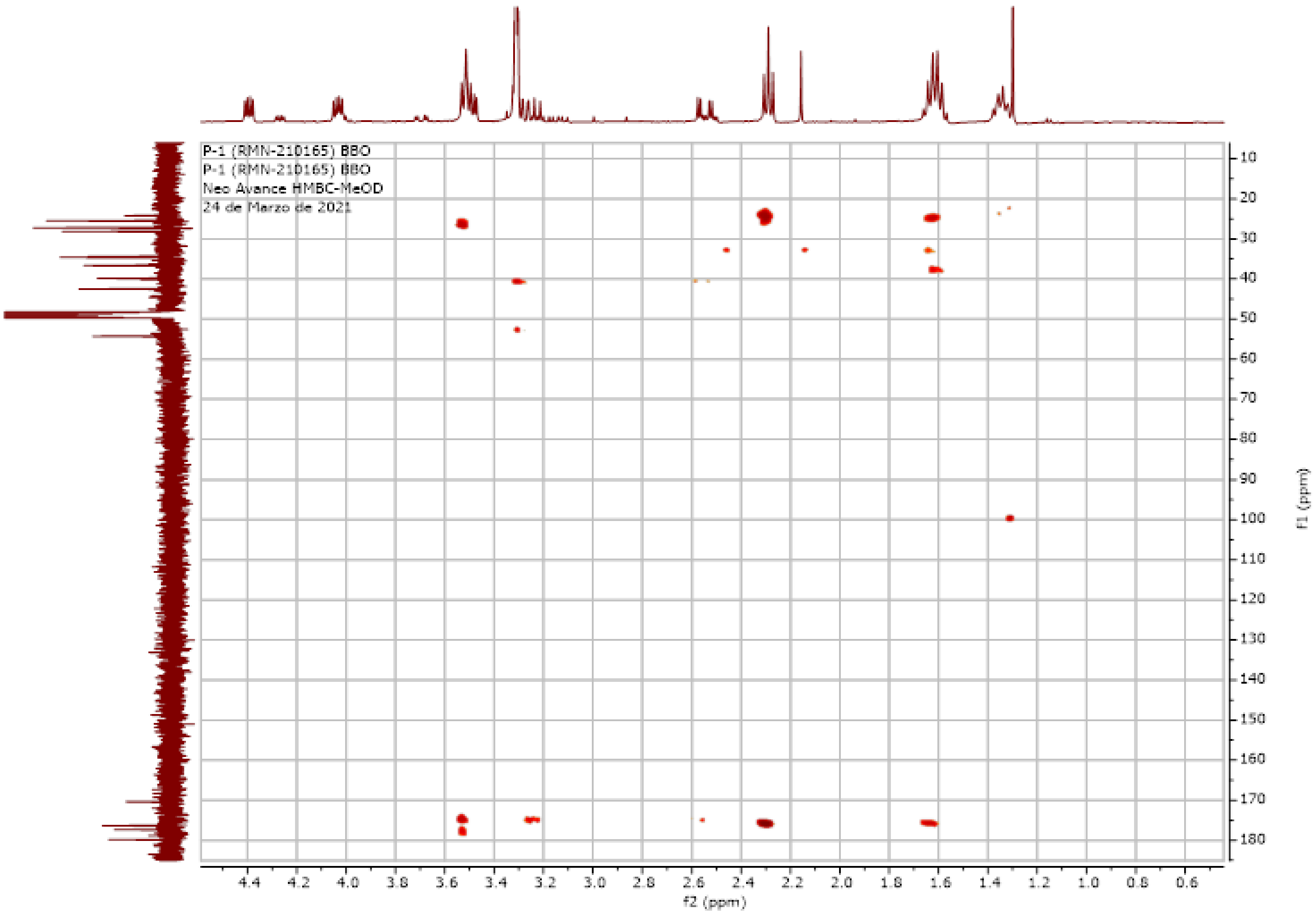

Figure S8. ^{1}H-NMR HMBC spectrum (400MHz, MeOD, 303 K) of compound **2.**

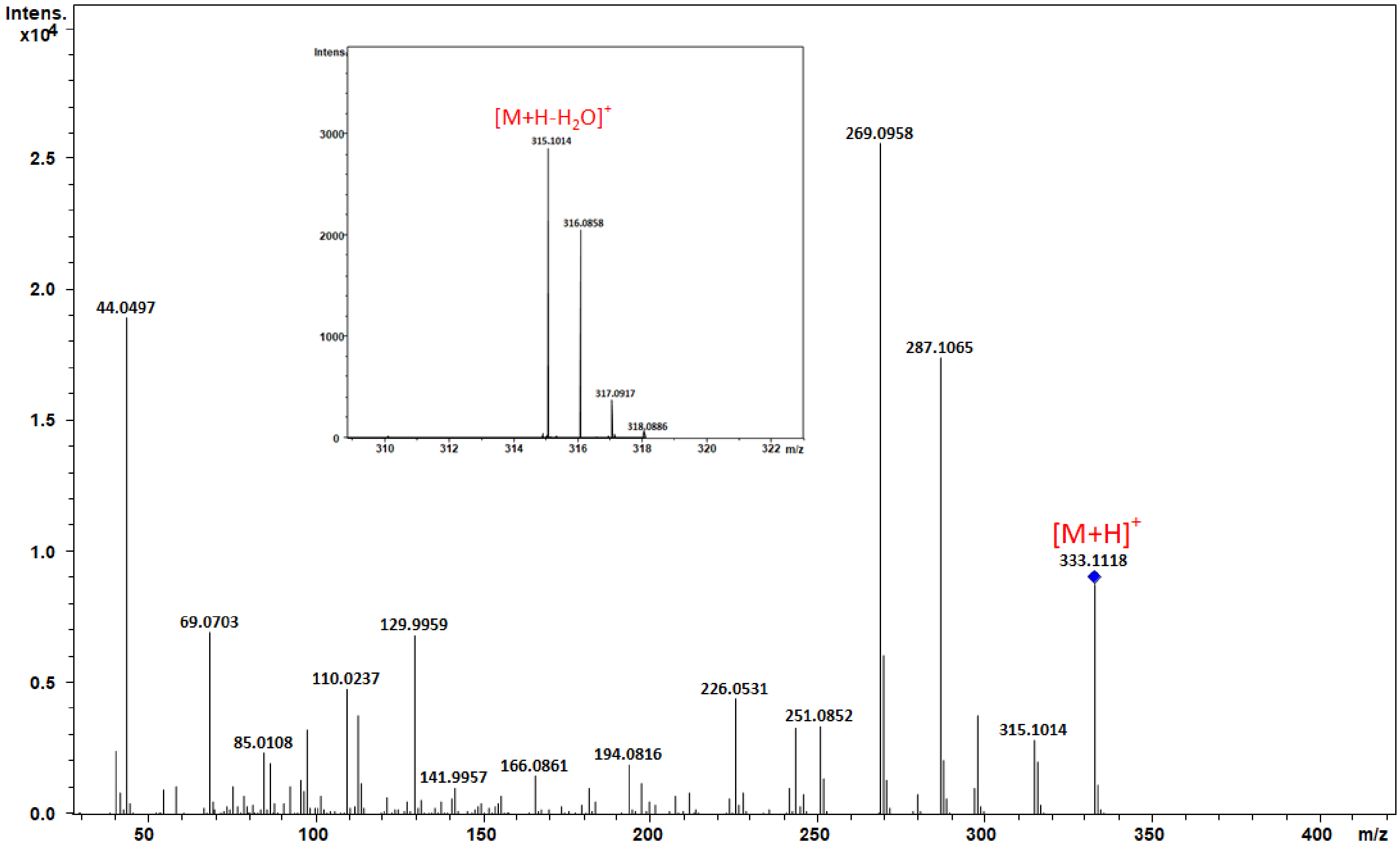

Figure S9. MS/MS for TIC of 4.6 min corresponding to compound **2**.

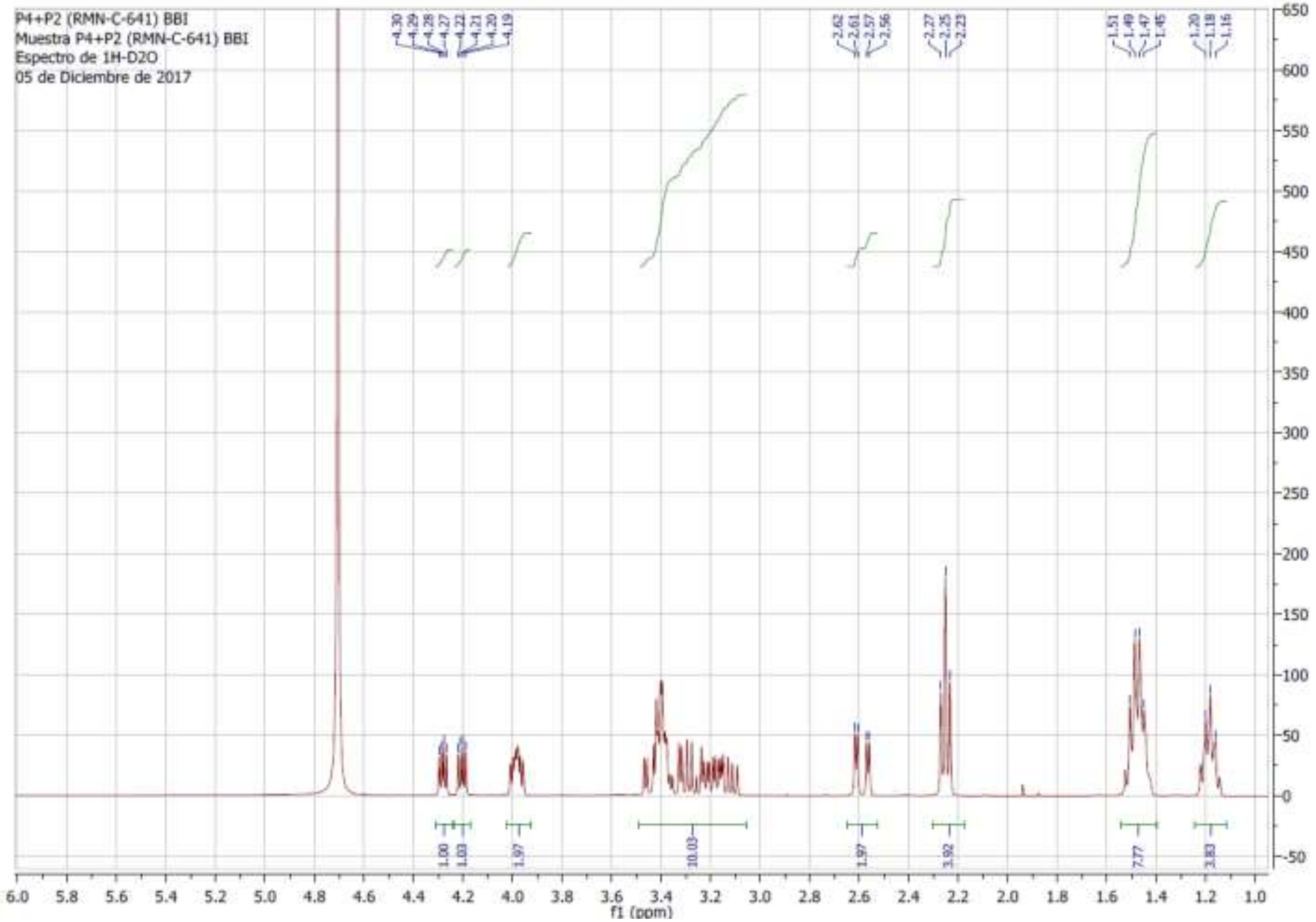

Figure S10. ^{1}H-NMR spectrum (400MHz, MeOD, 303 K) of mix products.
